# Syncopal Episodes of Arrhythmogenic Right Ventricular Cardiomyopathy in a Patient with Pre-existing Seizure Disorder

**DOI:** 10.7759/cureus.2760

**Published:** 2018-06-07

**Authors:** James R Kimber, Syed Rafay Ali Sabzwari, Hiwot Ayele

**Affiliations:** 1 Cardiology, Lehigh Valley Health Network, Allentown, USA; 2 Cardiology Fellowship, Lehigh Valley Health Network, Allentown, USA

**Keywords:** arrhythmogenic right ventricular cardiomyopathy, syncope

## Abstract

Arrhythmogenic right ventricular cardiomyopathy (ARVC), is a heritable condition that is an important, and under-recognized cause of sudden cardiac death. Microscopically, it is represented by fibrofatty replacement of myocardium involving the right ventricular inflow area, apex, and infundibulum. Common clinical manifestations of ARVC include palpitations, syncope, chest pain, dyspnea, and sudden cardiac death. This is a case of a 25-year-old male with a history of thalassemia, and tonic-clonic seizure status post head trauma with cystic encephalomalacia in left parietal lobe who described recurrent syncope. He was followed by neurology and maintained only on Lamotrigine. Episodes occurred within the span of four weeks and were without prodrome, lasting only a few seconds. On evaluation, blood pressure was 123/69 mmHg. Neurologic exam was grossly normal. Heart was regular rate and rhythm without gallops, murmur, or rub. An EKG showed normal sinus rhythm with an incomplete right bundle branch block and Epsilon waves in leads V1 and V2 without evidence of Brugada syndrome. The patient was admitted and had a 24-hour electroencephalogram that showed no seizure activity. A 2D Echo showed normal left ventricular function and no valvular disease. Eventual cardiac magnetic resonance imaging (MRI) showed small focal outpouchings of the right ventricular free wall. A diagnosis of ARVC was achieved, and the patient underwent electrophysiology (EP) study and successful implantation of a dual-chamber cardioverter defibrillator.

## Introduction

Arrhythmogenic right ventricular cardiomyopathy (ARVC), formerly known as arrhythmogenic right ventricular dysplasia (ARVD) is a heritable condition that is an important and under-recognized cause of sudden cardiac death. Histologically, it is represented by fibrous or fibrofatty replacement of myocardium involving the right ventricular inflow area, apex, and infundibulum, described in literature as the “triangle of dysplasia”. The clinical manifestations of ARVC most often include palpitations, syncope, chest pain, dyspnea, and sudden cardiac death. A diagnosis of arrhythmogenic right ventricular cardiomyopathy can be achieved with criteria established using the 2010 revised Task Force Criteria for the diagnosis of ARVC as defined by Marcus et al., 2010 [1]. These guidelines delineate into major and minor criteria, with a successful definitive diagnosis achieved with: any two major criteria, or one major and/or two minor criteria, or four minor criteria which describe global/regional dysfunction and structural alterations, tissue characterization of the wall, depolarization/conduction abnormalities, arrhythmia, and family history. Here, we describe a case of a 25-year-old male who presented with symptoms of recurrent syncopal events secondary to an overtly electrical arrhythmogenic right ventricular cardiomyopathy.

## Case presentation

The patient was a 25-year-old male who had a history of thalassemia minor as well as tonic-clonic seizures following head trauma with identified cystic encephalomalacia in the left parietal lobe. He was diagnosed with seizure disorder three years ago when he experienced episodes of dizziness with lightheadedness and sensation of spinning, with facial flushing. He was initially trialed on Levetiracetam, which seemed to increase the frequency of event rate and eventually was changed to Lamotrigine, which was his regular maintenance medication at the time of this admission.

**Figure 1:**
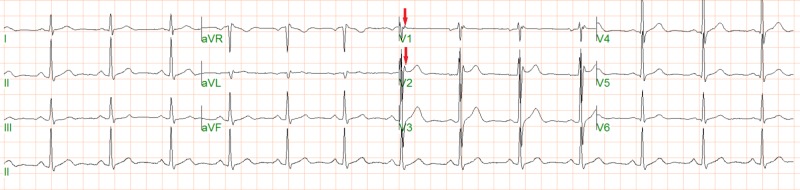
EKG showing incomplete right bundle branch block and epsilon waves (red arrow)

On initial presentation, he described nine episodes of syncope without prodrome occurring within the span of four weeks. He had loss of consciousness for approximately 20-30 seconds, usually witnessed. He did not experience any lightheadedness, dizziness, presyncope, palpitations, or tachycardia. His vitals were normal. On neurologic exam, cranial nerves were intact, sensation intact to light touch, reflexes intact bilaterally. Gait was normal and Romberg sign was negative. The cardiac exam revealed a regular rate and rhythm with normal S1 and S2 and without S3, S4, gallops, murmur, or rub. An EKG showed normal sinus rhythm with an incomplete right bundle branch block and Epsilon waves in leads V1 and V2 without evidence of Brugada syndrome (Figure 1). 

Laboratory data showed a hemoglobin level of 11.0 g/dL, white blood cell count 9.1 x 10^9^/L, sodium 140 mEq/L, potassium 4.1 mEq/L, and a point of care urinalysis was negative. A computed tomography (CT) scan of the head showed no intracranial hemorrhage or depressed skull fracture and stable cystic encephalomalacia in the left parietal lobe without infarction or intracranial mass.

Neurology consultation was requested and a continuous 24-hour video electroencephalogram (EEG) was performed, showing normal awake and sleep-prolonged video EEG without event. A lamotrigine level was checked and was therapeutic at 3.9 mcg/mL, and TSH was within normal limits. A urine drug screen was negative.

**Figure 2:**
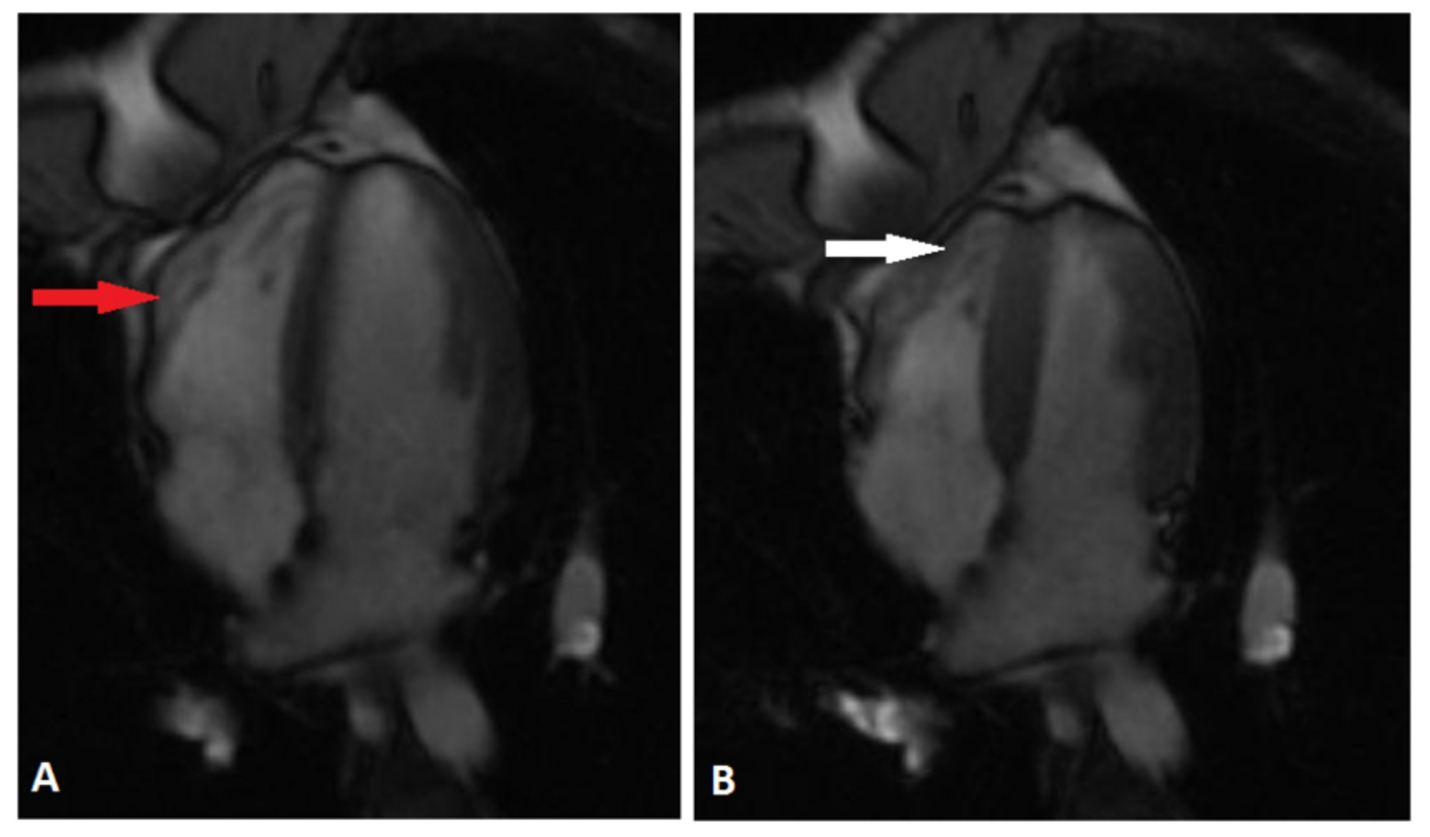
Magnetic resonance imaging (MRI) of heart with and without contrast showing aneurysmal dilatation of right ventricular free wall (red arrow) and focal outpouchings of the right ventricular free wall (white arrow); A-Diastole, B-Systole

Cardiology consultation was requested and patient was monitored on telemetry with no episodes of ventricular tachycardia. 2-D ECHO showed normal ventricular function and no valvular heart disease. A cardiac magnetic resonance imaging (MRI) was performed and showed left ventricular ejection fraction normal at 53% with the ventricular septum measuring 9 mm, posterior inferior wall measuring 10 mm and no evidence of abnormal late gadolinium enhancement. There were small focal outpouchings of the right ventricular (RV) free wall raising the question of micro-aneurysms, and with findings consistent with ARVC (Figure 2). The pericardium was normal.

A diagnosis of ARVC was achieved, and the patient underwent electrophysioloy (EP) study and successful implantation of a dual-chamber cardioverter defibrillator.

## Discussion

The differential diagnoses for ARVC are extensive, including Uhl’s anomaly (the partial or congenital absence of RV myocardium), cardiac sarcoidosis, Brugada syndrome, or RV outflow tract tachycardia secondary to structural heart disease. In this case, the patient achieved a diagnosis of ARVC satisfying two major criteria by 2010-revised criteria (RV akinesia by MRI, and epsilon wave in the right precordial leads) [1]. The presence of epsilon waves indicate a delay in right ventricular activation and are highly specific for ARVC [2].

ARVC has many potential etiologies and can be dysontogenetic with aberrant myocyte transdifferentiation, or due to degenerative conditions, infection such as coxsackie and those viruses causing myocarditis, inflammatory with rheumatologic disease, apoptotic, or genetic [3]. A familial inheritance to ARVC has been documented as early as 1982 and is now known to be predominantly autosomal dominant by inheritance. To date, seven genes have been described as contributing factors to this condition: JUP, D5P, PKP2, DSG2, DSC2, TGFβ, and TMEM43. These genes code for junctions involving cell-cell adhesion, specifically plakoglobin (JUP), desmoplaken (D5P), plakophilin-2  (PKP2), desmoglein-2 (DSG2), desmocollin-2 (DSC2) [4]. 

Additionally, a mutation in ryanodine receptor RYR2 is suspected in those patients with arrhythmia but lacking identifiable electrocardiographic or structural abnormalities [5]. Recessive forms of ARVC have also been described and are linked to extracardiac symptoms exemplified by Naxos disease and Carvajal syndrome, in which there is the woolly hair and keratodermia [4]. As ARVC often has a long-concealed phase, individuals with ARVC are at risk for sudden cardiac death, particularly during exercise. Therefore, suspicion for identification of patients with ARVC should prompt genetic analysis in first degree relatives.

This case was complicated by a history of seizure disorder, on maintenance medication, as well as prior history of head trauma and thalassemia. Thus, a complete neurologic evaluation had to be undertaken concordantly with cardiac evaluation of patient’s syncopal episodes. Of note, the patient had previously experienced pre-syncopal episodes for the past three years, having been attributed to partial-complex seizure disorder.

At the time of his original diagnosis, he had undergone extensive neurology evaluation, including a 24-hour ambulatory EEG, which did reveal frequent sharp waves and 2.5 Hz spike and wave complexes over the left frontal-anterior temporal region, interpreted as complex partial seizures versus non-epileptic event.

## Conclusions

In cases of syncope where epileptic seizure stands out as a strong differential, particular attention should be paid to the history, examination and EKG to consider possible cardiac etiology. ARVC is a heritable, important and often- missed etiology for ventricular arrhythmias that could also lead to syncope. Epsilon wave on precordial EKG leads, aneursymal right ventricular free wall with focal outpouchings on cardiac MRI are characteristic findings of ARVC that help confirm the diagnosis; an under-recognized cause of sudden cardiac death.
